# Ticks in the wrong boxes: assessing error in blanket-drag studies due to occasional sampling

**DOI:** 10.1186/1756-3305-6-344

**Published:** 2013-12-10

**Authors:** Andrew DM Dobson

**Affiliations:** 1 School of Biological and Environmental Sciences, University of Stirling, Cottrell Building, FK9 4LA, Stirling, UK

**Keywords:** Ticks, Sampling, Blanket-dragging, Bias, Error, Disease risk

## Abstract

**Background:**

The risk posed by ticks as vectors of disease is typically assessed by blanket-drag sampling of host-seeking individuals. Comparisons of peak abundance between plots – either in order to establish their relative risk or to identify environmental correlates – are often carried out by sampling on one or two occasions during the period of assumed peak tick activity.

**Methods:**

This paper simulates this practice by ‘re-sampling’ from model datasets derived from an empirical field study. Re-sample dates for each plot are guided by either the previous year’s peak at the plot, or the previous year’s peak at a similar, nearby plot. Results from single, double and three-weekly sampling regimes are compared.

**Results:**

Sampling on single dates within a two-month window of assumed peak activity has the potential to introduce profound errors; sampling on two dates (double sampling) offers greater precision, but three-weekly sampling is the least biased.

**Conclusions:**

The common practice of sampling for the abundance of host-seeking ticks on single dates in each plot-year should be strenuously avoided; it is recommended that field acarologists employ regular sampling throughout the year at intervals no greater than three weeks, for a variety of epidemiological studies.

## Background

Ixodid ticks are vectors of a profound range of infectious diseases of humans [1]. Biologists aiming to quantify either the situated epidemiological risk posed by ticks, or the relationship between tick abundance and environmental factors such as climate or host density, need a reliable method of estimating tick abundance. The relative immobility of ticks means that the traditional methods for sampling the abundance of winged insect vectors (using traps that attract with inducements such as dry ice) are of limited efficacy, especially for *Ixodes* spp. [2]. Instead, unfed ticks are typically sampled *in situ* by dragging a woollen blanket over the vegetation for a specified distance and counting the number of ticks, repeating the process a certain number of times in order to obtain replicate samples for each survey date *inter alia*[3-6].

Blanket-dragging is widely acknowledged to yield variable efficiency in different substrates [7,8], and even in the same substrates at different times of the year [9], since the blanket touches only the upper surface of the vegetation, the height of which may be seasonally variable [10]. Whatever the sampling regime, this method introduces poorly quantified biases. To give an extreme example, comparisons of spring/summer tick questing density in short grass plots and bracken (*Pteridium aquilinum*) plots are almost meaningless, given that a much smaller (and unknown) proportion of the questing population in bracken will come into contact with the blanket. Similarly, whilst a series of density indices from a bracken patch through the year in England, for example, will show a typical pattern of rising numbers in spring, a gradual decline through the summer and a small peak in the early autumn, the result will bear only a superficial resemblance to the true changes in tick numbers, given that the physical structure of the plant changes so markedly over the same period. The use of additional blankets of much narrower leading-edge width will compensate to a degree (for example by revealing the persistence of questing larvae after the growing bracken has lifted the larger blanket above larval questing height – see Figure seven in [9]), but there is little that can be done about the fact that the availability of questing positions increases dramatically during the spring (as the plant becomes taller and more structurally complex) and decreases equally dramatically during the autumn as the plant dies back. Drags of a full-size (typically c.1 m^2^) blanket from early summer to late autumn will tend therefore to ‘capture’ a much smaller proportion of questing ticks of all stages than the same drags at other times of the year. This sort of bias may be anticipated, but so far can only be calibrated in a qualitative fashion. Specific tests involving blanket-drags of plots with different substrates have not yet been published, though some preliminary studies have been carried out (L. Gilbert, pers. comm.).

A different type of error may arise from the temporal regime employed in blanket-drag studies. It is a frequent practice in the acarological literature to sample on just one or two dates annually at each plot of interest, making sure that the sample dates are within a two-month period (for example) aligned with the known period of peak tick activity. This practice ignores the fact that the seasonality of tick questing behaviour in temperate regions has profound implications for the representativeness of any single sample. Furthermore, ticks in different plots within the same square kilometre may display strongly contrasting questing patterns, and each plot may yield substantial between-year variation in phenology (e.g. the timing of the spring peak for any stage) [9]. In this paper, I attempt to demonstrate the potential for error in the practice of selecting either one or two sampling dates during a window of assumed peak tick activity, by re-sampling from an existing higher-resolution dataset of nymphal tick activity and comparing the results of different sampling regimes.

## Methods

### Study sites and data collection

Nymphal tick abundance data considered for use in this study were collected throughout 2008 and 2009 by three-weekly blanket-dragging at 20 plots spread across three sites in southern England (Webber’s Post, Exmoor National Park, Somerset; Wilverley, New Forest, Hampshire; Richmond Park, London). Locations and data collection protocols are fully described in [9]. In order to reduce any bias associated with the stochasticity of small samples, only the five plots with the highest recorded tick activity, as measured as an average across sampling dates throughout the two years, were considered for the current study. Briefly: plots 1–3 are from Exmoor, and 4–5 from the New Forest; plot 1 - mixed *Vaccinium* spp./heather in mixed woodland; plot 2 - vegetated mixed woodland floor; plot 3 – *Vaccinium* spp. in *Pinus* spp. woodland; plot 4 – grassy path edge in mixed woodland; plot 5 – bracken in mixed woodland. No plots from the Richmond Park site were selected, as tick density was relatively low.

### Interpolation and re-sampling

The aim was to create a set of realistic daily tick data from which ‘samples’ could be taken in such a way as to simulate field procedures. The three-weekly samples are obviously not suitable for this purpose in their raw form, so they were transformed into daily series by simple linear interpolation between the actual sample dates. These new data series (one for each plot) could then be treated as the ‘real’ data, and a sampling visit could be simulated by picking a date and looking up the corresponding data value.

The interpolated series were re-sampled in ways that simulate (i) sampling on a single date during a period of assumed peak activity, (ii) sampling on two dates, 30 days apart, during a period of assumed peak activity, (iii) sampling every three weeks. The last of these provides a realistic baseline against which to compare the first two. Note that the general approach opens the opportunity of a very misleading bias if the original field data were used to represent three-weekly sampling, since they would be artificially accurate; this explains why three-weekly samples were re-estimated, even though they already existed in empirical form.

(i) To simulate the practice of taking a single sample in a two-month period, the date of peak recorded activity in 2008 at each of the five study plots was recorded. The earliest date within the 61-day window centred on the peak date was then selected, and 366 was added so that the date would fall in 2009 (note that 2008 was a leap year). The nymph abundance value in the model (interpolated) series for this date was recorded. This process was repeated for the remaining 60 dates, providing, for each plot, 61 possible alternative samples in 2009 based upon the peak recorded date in 2008 at that plot.

(ii) To simulate double samples (i.e. two samples taken within a month of one another during the assumed period of peak activity), the first 31 dates as described above were selected (up to and including the peak date), to which 30 days were also added to provide the second dates. Samples from the 2009 model series were taken on these paired dates and averaged, yielding 31 possible samples.

(iii) To judge the efficiency of single and double samples against that of three-weekly samples, the model series were re-sampled at three-weekly intervals using all 21 possible start dates, generating the full range of possible three-weekly data series. The peak nymphal abundances for each of these 21 samples were then calculated.

The process outlined above was designed to simulate the errors that might arise if the peak activity period for any given plot in year *N* is taken to be the same as the peak in year *N*-1. Also simulated was the error that might arise if peak activity in year *N* is assumed to be the same as the peak in year *N*-1 at a separate, nearby plot. For this scenario, the single and double sample dates were generated using 2008 model data from plot 1, but applied to 2009 model data in plots 2 and 3, both of which were both within 300 m of plot 1 (in Exmoor National Park).

The extent to which three-weekly samples accurately characterise the model data was assessed by comparing the 21 three-weekly samples for each plot described above with the model data series for those plots. For each of the 21 samples, a mean value was computed, giving 21 annual values for each plot. The distribution of mean values for each plot was compared with the single mean value of the model data for each plot. This measure is hereafter referred to as the AUTG – or Area Under The Graph of tick abundance against time.

Significance testing on the differences between means of single, double and three-weekly samples is not carried out; the 61 and 31 replicates of the single and double samples, respectively, are not equivalent to numerous replicates taken by a single fieldworker, but instead represent numerous possible values resulting from the single or double sample that the fieldworker might have taken. This study is designed to assess the maximum possible degrees of error resulting from certain sampling regimes; the mean and median are thus no more instructive than any individual data-point – all are equally likely to be the single (or double) value that the hypothetical fieldworker records. (Note that there is a distinct difference between taking repeated samples on just one or two days, and taking repeated samples at regular intervals; here a ‘single sample’ stands for the mean of any number of replicated blanket drags carried out on a single day). For this reason, the graphical results show only data ranges, not means, deviances or inter-quartiles.

All data manipulation and graphical output was carried out in R version 3.0.1 [11].

## Results

### Peak activity – 2009 sampling directed by 2008 peak

**Figure 1:**
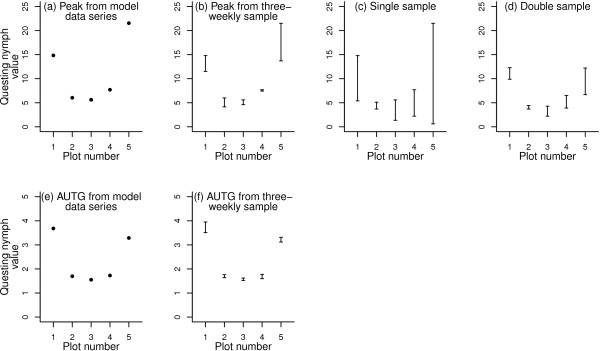
**Recorded and resampled estimates of peak and total nymphal tick abundance in 2009 at five plots in southern England.** For each plot: Graph **(a)** shows the peak nymphal activity in 2009 (model data created by daily interpolation of three-weekly tick data collection); graph **(b)** shows the ranges of peak nymphal activity values for the 21 possible three-weekly sampling regimes of the model data for each plot; graph **(c)** shows ranges of activity values taken on all possible single-sample dates within a 61-day window centred on the recorded peak at each plot the previous year; graph **(d)** shows ranges of activity values taken on all 31 possible double-sample dates within the same 61-day window; graph **(e)** shows the area under the graph (AUTG – see text) values of the model data series; graph **(f)** shows the distribution of AUTG values for the three-weekly sampling regimes of the model data.

Single samples varied greatly between plots in their precision in describing the peak nymphal activity (Figure 1c). Low variability (e.g. plot 2) implies a relatively broad peak, such that a wide spread of sample dates yields similar activity values, whilst high variability (e.g. plot 5) implies a more sharply delineated peak. The latter sort of activity profile leads to the greatest potential error when taking only single annual samples. In all but one plot (plot 2), three-weekly sampling (Figure 1b) yielded more precise estimates (i.e. with a smaller range of possible values) of peak activity values than single sampling. The double samples (Figure 1d) were far more precise than single samples, and slightly more so than three-weekly samples in estimating peak activity, but there was still considerable overlap between the data ranges of all plots, even in plots 4 and 5, where the model data reveal that the peak in the latter is actually three times higher than that of the former (Figure 1a). The three-weekly samples tended to be closer to the ‘real’ peak value.

### Peak activity – 2009 sampling directed by peak at adjacent plot

**Figure 2:**
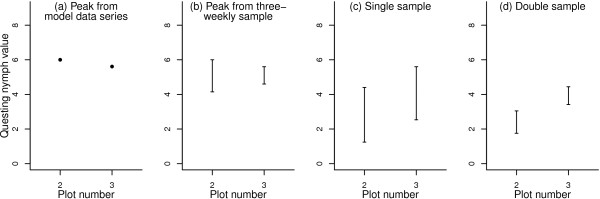
**Recorded and resampled estimates of peak and total nymphal tick abundance in 2009 at two plots in southern England.** Graph **(a)** shows the peak of recorded activity in 2009; graph **(b)** shows ranges of all possible three-weekly samples; graph **(c)** shows ranges of nymphal activity values taken on all possible single-sample dates within a 61-day window centred on the recorded peak at a separate plot the previous year; graph **(d)** shows ranges of activity values taken on all 31 possible double-sample dates within the same 61-day window.

**Figure 3:**
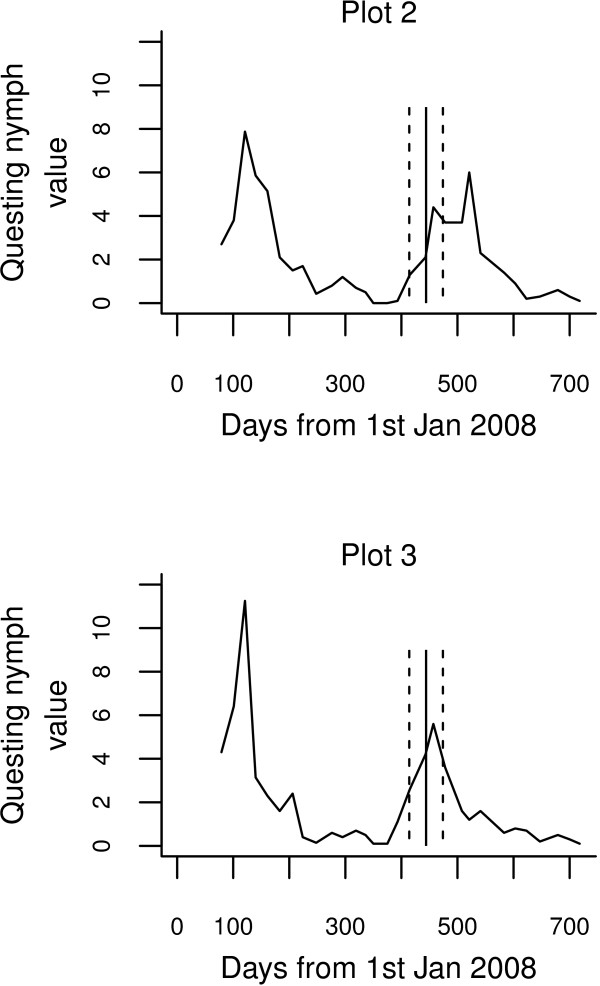
**Recorded nymphal tick activity from three-weekly sampling visits at two plots in Exmoor, south-west England.** The solid vertical line marks the date at which the peak nymph activity was recorded the previous year at a separate nearby plot; dashed lines denote the limits of the 60-day sampling window from which the samples shown in Figure 2c and d were taken.

When the 2008 peak at plot 1 was used to produce a sampling window for plots 2 and 3, the single samples produced much larger data ranges than either double samples or three-weekly samples (Figure 2). Variability in the double sample was similar to that in the three-weekly sample, but across the full range of values, the double sample ranked the two plots incorrectly, whilst the data ranges from the three-weekly sample overlapped completely and were closer to the ‘real’ values. Figure 3 shows the timing of peaks in 2009 at plots 2 and 3, with the sampling window superimposed; plot 3′s 2009 peak is better captured than plot 2′s 2009 peak by the peak at plot 1 in 2008. This temporal mismatch accounts for the relatively low estimate of the peak at plot 2 in single and double samples.

### Peak vs. AUTG

Comparison of Figure 1a with 1e indicates how the peak abundance may not accurately reflect relative risk of tick exposure across plots; plot 5 has the highest peak but plot 1 has the highest activity throughout the year. Figure 1f demonstrates that the overall risk – the AUTG – is very adequately represented by three-weekly sampling.

## Discussion

Accurate estimation of the abundance of host-seeking ticks must underpin any study seeking to quantify and/or explain the risk of tick-borne disease. This study demonstrates how occasional annual sampling during periods of assumed peak activity may lead to heavily biased estimates of tick abundance.

A variety of factors may combine to influence questing phenology. Whilst the onset of questing activity is linked to air temperature and may therefore be regionally co-ordinated [12-14], temperature will be strongly mediated by habitat, such that ticks in adjacent woodland and field-edge plots (for example) will experience very different microclimates, such that the peaks of activity differ in timing [15]. Apparent variation between years may also be caused by aggregated distributions – especially of larval ticks – that render even replicated samples vulnerable to stochastic peaks or troughs that mask true patterns.

The present study illustrates the potential for considerable variation in phenology even in nearby plots experiencing very similar conditions. The 2008 peaks at plots 2 and 3 were on the same day (though of course, the resolution of the sampling window means that we can only be sure that the peaks were within 21 days of one another), but they were 64 days apart in 2009 (an advance of 29 days at plot 3 and a delay of 35 days at plot 2). By contrast, the recorded peaks at plot 1 were one day apart in the two years. All three plots were in sheltered woodlands at the same elevation within 300 m of each other.

It follows that any attempt to discover relationships between any given environmental factor and tick questing density may be hampered at the outset if occasional sampling is used to compare tick density at different plots, since without knowledge of the precise phenology of each site and in each year, the samples will represent variable and wholly unknown proportions of the maximum questing density. Indeed, it can be said with some confidence that single samples within assumed periods of peak activity are wholly unsuitable for tick abundance studies. Any such study, justified by statements about ‘known tick activity periods in the area’ should be viewed with a great deal of scepticism. Double samples within a window of two months constitute a considerable improvement, but for measures of overall risk of tick contact, there is no substitute for regular sampling throughout the year, with a sampling interval of no more than three weeks.

This study is based upon two sites in southern England, meaning that the outcome will be applicable in other regions to varying degrees. In an important sense, however, the representativeness of these sites is immaterial – precisely *because* it remains unknown. Without a high-resolution, multi-annual dataset, the fieldworker will be unable to tell whether or not the potential for error applies on their sites. He or she thus faces a Catch-22 situation – the only way to avoid requiring a three-weekly (or better) sampling regime is to establish that it isn’t required, and the only way to do so is to employ a three-weekly (or better) sampling regime.

Even a regular sampling regime can be expected to ‘miss’ periods of peak activity in an unknown and variable manner across years and plots. Peak abundance is therefore a parameter that, whilst often presented, must only rarely be measured with any accuracy. Difficulties of measurement aside, there are good reasons to be wary of using peak abundance as an informative parameter. Consider two identical tick populations in adjacent plots experiencing identical host contact rates but different microclimates (mediated by habitat, for example). A greater degree of vegetative shading in the first plot could cause a relatively slow spring emergence of active ticks, thereby producing a later, lower peak. A comparison of peak questing abundance, even if accurately judged in terms of timing, would suggest a difference in population size between the two that did not exist. By contrast, the measure AUTG – the area under the graph of tick abundance against time – provides a single metric describing overall risk of exposure to ticks throughout the year, and this study demonstrates that three-weekly sampling allows a very accurate and precise estimation of AUTG. There was a broad alignment in the present study between peak recorded abundance and AUTG (though a larger selection of plots would have clarified the nature of the relationship), but the ordering of the plots was nonetheless dependent upon the metric used; peak and AUTG values were thus not directly equivalent. Regular sampling also accommodates the fact that humans are not active merely at times of maximum tick questing activity; indeed, overall human exposure to ticks might well peak in late summer, during school holidays, after the main period of nymphal activity in many parts of Europe and North America.

Long-term, high-resolution tick activity datasets are time-consuming (and therefore often expensive) to generate, and are far less common than perhaps they ought to be; for example, in the highlands of Scotland, where cases of human infection with *Borrelia burgdorferi* s.l. are presented at a rate of 28 per 100,000 of the population [16], questing phenology data have not yet been published. Unfortunately, single annual samples appear to be an ineffective shortcut.

## Conclusion

In general, the literature dealing with the effects of biotic and abiotic conditions on tick density is split between studies that provide detailed phenological profiles of tick activity and those that use greatly simplified metrics derived from isolated sampling events assumed to coincide with activity peaks. However, the complexities of tick biology render such latter simplifications vulnerable to significant errors; we advocate the former approach of multiple samples taken at regular intervals in order to accurately characterize tick population patterns.

## Competing interest

The author declares that he has no competing interests.
